# The Alginate and Motility Regulator AmrZ is Essential for the Regulation of the Dispersion Response by *Pseudomonas aeruginosa* Biofilms

**DOI:** 10.1128/msphere.00505-22

**Published:** 2022-11-14

**Authors:** Manmohit Kalia, Matthew D. Resch, Kathryn E. Cherny, Karin Sauer

**Affiliations:** a Department of Biological Sciences, Binghamton University, Binghamton, New York, USA; b Binghamton Biofilm Research Center, Binghamton University, Binghamton, New York, USA; University of Iowa

**Keywords:** AmrZ, BdlA, PA1891, biofilm matrix, c-di-GMP, dispersion, endonuclease, hydrolase, hyperdispersive, *napB*, regulation

## Abstract

Dispersion is an active process exhibited by Pseudomonas aeruginosa during the late stages of biofilm development or in response to various cues, including nitric oxide and glutamate. Upon cue sensing, biofilm cells employ enzymes that actively degrade the extracellular matrix, thereby allowing individual cells to become liberated. While the mechanism by which P. aeruginosa senses and relays dispersion cues has been characterized, little is known about how dispersion cue sensing mechanisms result in matrix degradation. Considering that the alginate and motility regulator AmrZ has been reported to regulate genes that play a role in dispersion, including those affecting virulence, c-di-GMP levels, Pel and Psl abundance, and motility, we asked whether AmrZ contributes to the regulation of dispersion. *amrZ* was found to be significantly increased in transcript abundance under dispersion-inducing conditions, with the inactivation of *amrZ* impairing dispersion by P. aeruginosa biofilms in response to glutamate and nitric oxide. While the overexpression of genes encoding matrix-degrading enzymes *pelA*, *pslG*, and/or *endA* resulted in the dispersion of wild-type biofilms, similar conditions failed to disperse biofilms formed by dt*amrZ*. Likewise, the inactivation of *amrZ* abrogated the hyperdispersive phenotype of PAO1/pJN-*bdlA*_G31A biofilms, with dt*amrZ*-impaired dispersion being independent of the expression, production, and activation of BdlA. Instead, dispersion was found to require the AmrZ-target genes *napB* and PA1891. Our findings indicate that AmrZ is essential for the regulation of dispersion by P. aeruginosa biofilms, functions downstream of BdlA postdispersion cue sensing, and regulates the expression of genes contributing to biofilm matrix degradation as well as *napB* and PA1891.

**IMPORTANCE** In P. aeruginosa, biofilm dispersion has been well-characterized with respect to dispersion cue perception, matrix degradation, and the consequences of dispersion. While the intracellular signaling molecule c-di-GMP has been linked to many of the phenotypic changes ascribed to dispersion, including the modulation of motility and matrix production, little is known about the regulatory mechanisms leading to matrix degradation and cells actively leaving the biofilm. In this study, we report for the first time an essential role of the transcriptional regulator AmrZ and two AmrZ-dependent genes, *napB,* and PA1891, in the dispersion response, thereby linking dispersion cue sensing via BdlA to the regulation of matrix degradation and to the ultimate liberation of bacterial cells from the biofilm.

## INTRODUCTION

Biofilms are communities or aggregates of bacterial cells enclosed in a self-produced polymeric matrix (1). The ability to form a biofilm is a common trait of a diverse array of microbes (2). Relative to their free-living, planktonic counterparts, the biofilm mode of growth affords bacteria protection from pH changes, exposure to oxygen radicals, biocides, and antimicrobial agents (3), and the benefit of remaining stationary within a favorable environmental niche or host. However, biofilms have also evolved mechanisms by which to escape the sessile growth mode when needed. One of these mechanisms is referred to as dispersion, and it involves bacterial cells actively liberating themselves from matrix-encased biofilms and reverting to the planktonic mode of growth (4–7). Dispersion is apparent by single cells actively escaping from the biofilm, leaving behind eroded biofilms and microcolonies with central voids (4–13).

While additional mechanisms resulting in the disaggregation of biofilms are known (14), two types of active dispersion mechanisms have been reported: native dispersion and environmentally induced dispersion. Little is known about native dispersion; however, evidence suggests that in Pseudomonas aeruginosa, native dispersion occurs in response to a self-synthesized signaling molecule, the fatty acid molecule cis-2-decenoic acid (8, 15, 16). In contrast, environmentally induced dispersion occurs in response to sensing factors present in or changing conditions of the surrounding environment. Examples of dispersion cues include changes in oxygen and nutrient availability and the presence of noxious compounds, such as heavy metals or nitric oxide (17–26).

Environmental dispersion cues have been reported to be perceived and relayed by membrane-bound sensory proteins. In P. aeruginosa, the detection of sugars and amino acids has been linked to the membrane-bound diguanylate cyclase NicD, which belongs to a family of seven transmembrane (7TM) receptors (9, 27), whereas nitric oxide has been reported to be perceived by NbdA (28), an MHYT domain-harboring phosphodiesterase, although heme-nitric oxide/oxygen-binding (H-NOX) domain proteins have been reported in other species to contribute to nitric oxide sensing (29–33). MHYT domains consist of six transmembrane domains, three of which contain the conserved amino acid residues methionine, histidine and tyrosine after which this domain is named. A signal relay subsequently involves the activation of the chemotaxis transducer protein BdlA (9, 12, 27). Activation requires phosphorylation and temporarily elevated c-di-GMP levels, resulting in nonprocessive proteolysis and the activation of BdlA (9, 12, 27). BdlA, in turn, activates the phosphodiesterase DipA and recruits a second phosphodiesterase RbdA to ultimately reduce cellular c-di-GMP levels (9, 12). An additional player is the diguanylate cyclase GcbA, which contributes to BdlA cleavage during biofilm growth (12, 13), with the inactivation of *gcbA* impairing BdlA activation and dispersion (12, 13). Collectively, dispersion cue perception and the subsequent relay coincide with dispersed cells being characterized by decreased levels of the intracellular signaling molecule c-di-GMP, relative to biofilms (9–11, 13, 19, 28, 34–36). A consequence of dispersion is bacteria leaving the biofilm structure (37). Biofilms are enmeshed in a polymeric matrix. In P. aeruginosa, the major components of the biofilm matrix are the polysaccharides Pel, Psl, and alginate, as well as extracellular DNA (eDNA) and proteins (38, 39). Given that biofilm cells have to liberate themselves from the enmeshed biofilm structure during dispersion, it is not surprising that dispersed cells demonstrate both an increased release of matrix-degrading enzymes, and an increased expression of genes encoding matrix degrading enzymes, including the endonucleases EndA and EddA as well as the glycoside hydrolases PelA and PslG (34, 40–42). Moreover, the overexpression of *endA*, *eddA*, and *pelA* by P. aeruginosa biofilms coincided with dispersion events (34, 40, 41). The finding of biofilm matrix degradation playing a major role in dispersion is supported by the exposure of P. aeruginosa biofilms to purified hydrolases, including PelA and PslG, resulting in the disassembly of the biofilms (43–45). In contrast, while eDNA plays a significant role in the biofilm matrix by providing stability and structure (38, 46–48), exogenously added DNases have only been able to disassemble young (but not mature) P. aeruginosa biofilms (49) and biofilms by species other than P. aeruginosa, such as P. putida, Staphylococcus aureus, Shewanella oneidensis, and Bacillus licheniformis (38, 46, 47, 50–53).

While much is known about dispersion cue perception (9, 11, 16, 28, 54), the release from the matrix-enclosed biofilm structure (34, 37, 40, 41, 55), and the consequences of dispersion (43), little is known about the regulatory mechanisms leading to matrix degradation and to cells actively leaving the biofilm. In P. aeruginosa, regulatory systems capable of modulating the intracellular level of c-di-GMP, motility, and matrix production include the HptB (56–59), Wsp (60, 61), Pil-Chp (62–64), and SadBC/BifA (65, 66) systems, as well as AmrZ (67) and FleQ, and even, to some extent, the Psl polysaccharide itself (68, 69). Among these, the transcription factor alginate and motility regulator Z (AmrZ) stands out, as it directly or indirectly affects several genes encoding components and/or phenotypes previously linked to dispersion. For example, AmrZ has been reported to modulate the abundance of Psl in biofilms, with an *amrZ* mutant expressing large amounts of Psl exhibiting a hyperaggregative phenotype. The effect of *amrZ* inactivation on biofilm biomass accumulation appears to be less consistent, with Jones et al. (67) reporting an *amrZ* mutant forming hyperbiofilms, relative to wild-type biofilms, while Jones et al. (70) noted an *amrZ* mutant forming biofilms that featured large microcolonies that exceeded those of wild-type biofilms without affecting the overall biofilm biomass. However, the phenotypes associated with *amrZ* inactivation, including those with increased Psl production and hyperaggregation, are commonly associated with the accumulation of c-di-GMP. Moreover, AmrZ represses the diguanylate cyclase-encoding gene *gcbA* (PA4843) (67), the transcription and motility of *fleQ* (67, 71, 72), and the production of the extracellular polysaccharide Psl (67, 70) while activating alginate production (73) and twitching motility (67, 74). RNA-seq and ChIP-seq further indicated that AmrZ affects the expression of *endA*, *eddA*, and *cdrA* (67). While each of these AmrZ-regulated genes and phenotypes have been linked to biofilms and P. aeruginosa pathogenicity, with AmrZ reciprocally regulating motility and matrix production, no link to dispersion has been reported. Therefore, the goal of this study was to investigate the involvement of AmrZ in the induced dispersion response of P. aeruginosa biofilms.

## RESULTS

### Inactivation of *amrZ* impairs the dispersion response.

Biofilm dispersion has been reported numerous times to coincide with cells liberating themselves from the biofilm matrix and returning to the planktonic mode of growth, and this is apparent by dispersed cells demonstrating enhanced expression of flagella genes, enhanced expression of genes encoding matrix degrading enzymes, including *pelA*, *pslG*, *endA*, *eddA*, and *eddB*, but reduced expression of pili genes (11, 40, 41).

As AmrZ has been reported to reciprocally regulate matrix production and motility, we first explored whether AmrZ is required for dispersion by P. aeruginosa biofilms in response to the known dispersion cues, glutamate and nitric oxide. We reasoned that if AmrZ contributes to dispersion, then the inactivation of *amrZ* would render P. aeruginosa biofilms dispersion-deficient. We made use of the dt*amrZ* mutant strain, which was previously reported by Jones et al. (67, 70) to be affected in the expression of extracellular polysaccharides and to form biofilms composed of taller microcolonies, compared to those of wild-type biofilms.

Biofilms by P. aeruginosa PAO1 and dt*amrZ* were grown in biofilm tube reactors under flowing conditions for 5 days, and this was followed by a sudden exposure of the biofilms to dispersion cues, namely, glutamate and SNP as a source of nitric oxide (NO). Biofilm effluents were collected, and the absorbance values of the effluents were subsequently determined at 600 nm. Dispersion events were apparent by sharp increases in the absorbance values of the effluent within 15 to 20 min upon the induction of dispersion, compared to untreated biofilms, as determined using tube reactors (9, 11, 12, 17, 27, 28).

**FIG 1:**
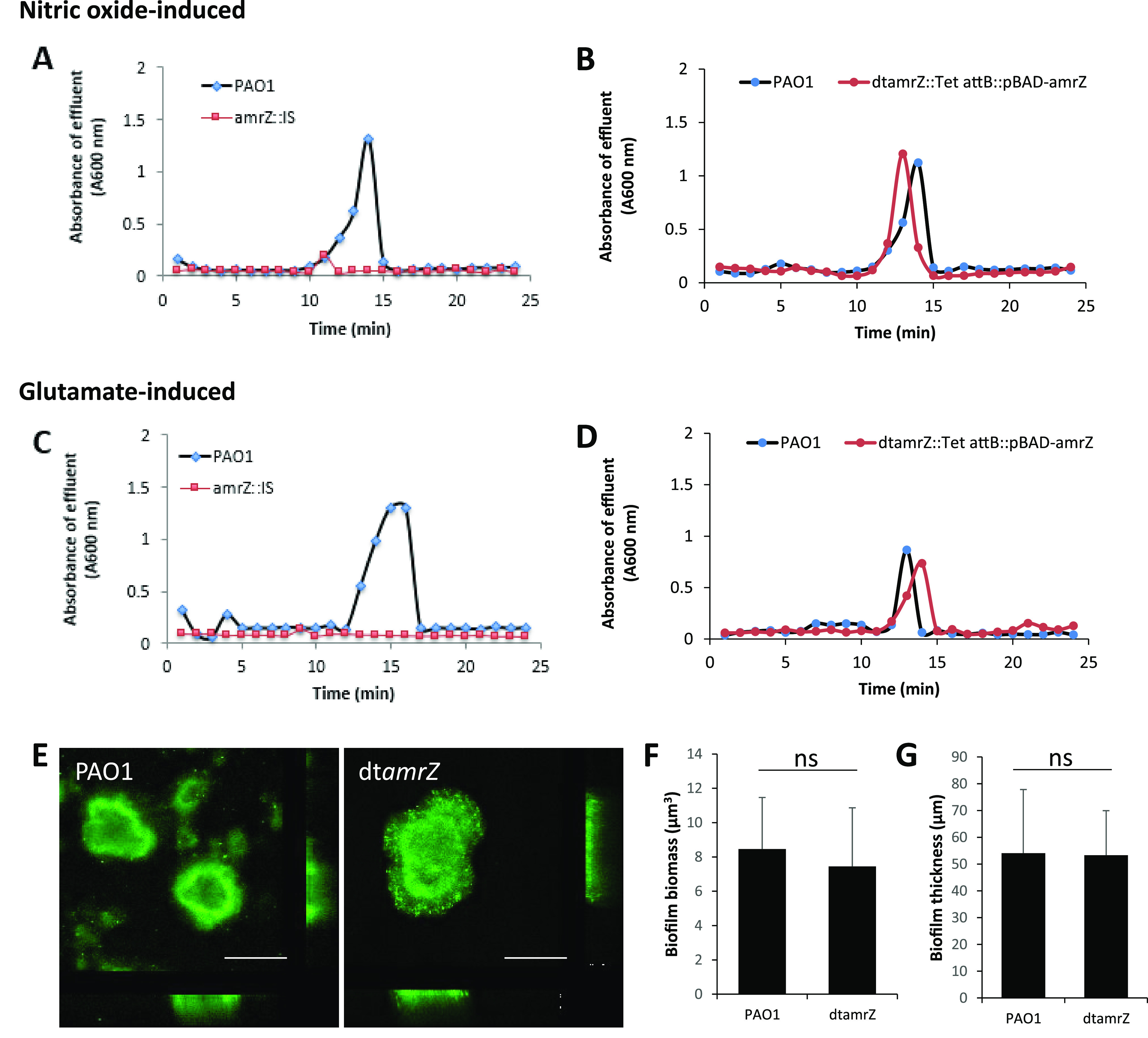
Dispersion of P. aeruginosa biofilms is dependent on AmrZ. Biofilms were grown in 5-fold diluted VBMM in continuous flow biofilm tube reactors. Dispersion was induced after 5 days of growth via the addition of (A and B) sodium nitroprusside (as a source of nitric oxide) or (C and D) glutamate to the growth medium. The effluent from tube reactors was collected for 24 min at 1 min intervals, and the absorbance was determined by spectrophotometry at 600 nm. Brief spikes in the absorbance of the effluent are indicative of positive dispersion responses. The dispersion of biofilms formed by dt*amrZ* mutants (A and C) and chromosomally complemented (B and D) was assessed relative to that of wild-type biofilms. The chromosomally complemented strain was grown with 0.1% arabinose to induce the expression of *amrZ*. The plots shown are representative of at least 3 biological replicates, each of which consisted of 8 technical replicates. (E) Representative confocal images of biofilms formed by the indicated strains, grown for 5 days under continuous flow conditions. Scale bar, 100 μm. COMSTAT was used to quantitatively determine the biofilm biomass (F) and biofilm thickness (G) of PAO1 and the dt*amrZ* mutant strains. ns, not significant, as determined using a Student’s *t* test.

While wild-type biofilms dispersed in response to glutamate and NO, which was apparent by a sharp increase in the absorbance values of the biofilm effluents, biofilms formed by dt*amrZ* failed to do so (Fig. 1A and C). In contrast, biofilms formed by a dt*amrZ* mutant strain that was chromosomally expressing wild-type *amrZ* dispersed in response to glutamate and NO (Fig. 1B and D).

To ensure that the lack of dispersion by the *dtamrZ* mutant strain was not due to a lack of biofilm formation, we quantitatively analyzed the biofilm architecture of the respective mutant strain via confocal microscopy and COMSTAT. Under the conditions tested, dt*amrZ* formed biofilms that were, overall, similar in architecture to biofilms formed by PAO1 (Fig. 1E), with a COMSTAT analysis confirming dt*amrZ* forming biofilms as comparable to wild-type biofilms (Fig. 1F and G). Our findings regarding the dt*amrZ* forming biofilms that were similar to the wild-type are in contrast to previous reports of this mutant strain forming hyperbiofilms (67); however, visual observations of the biofilm architecture support dt*amrZ*, in agreement with reports by Jones et al. (70), to form slightly larger microcolonies, compared to those of PAO1 (Fig. 1E). The difference in biofilm architecture is likely due to differences in the age of the biofilms, with Jones et al. having analyzed 24-hour-old, flow cell grown biofilms, whereas this study made use of 5-day-old, flow cell grown biofilms.

### Induction of *amrZ* expression does not lead to dispersion.

**FIG 2:**
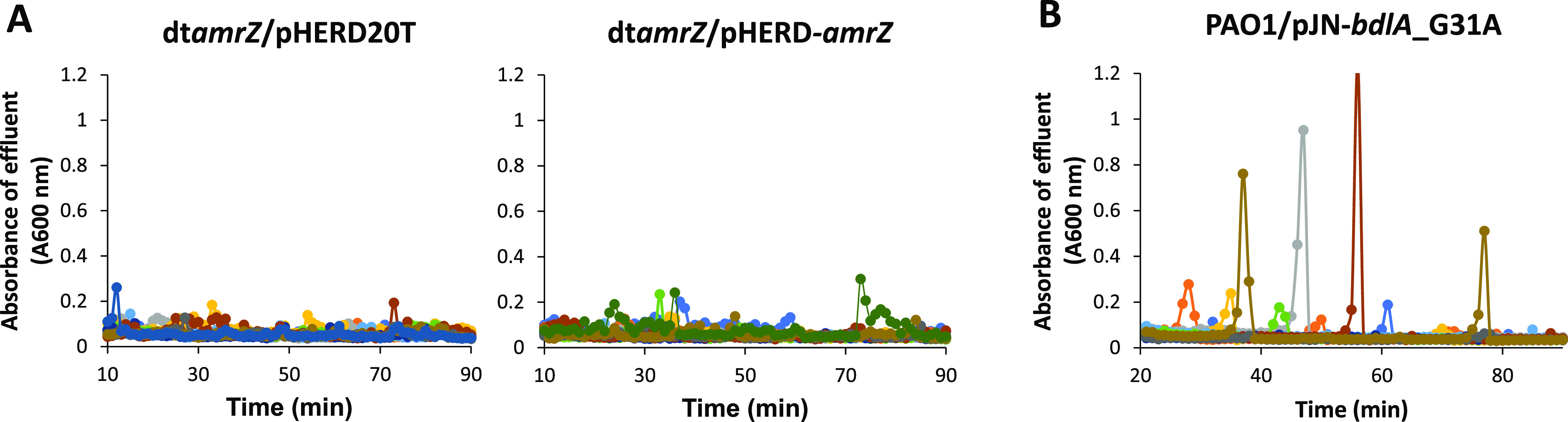
Overexpression of *amrZ* does not coincide with dispersion events. (A) Biofilms formed by dt*amrZ* harboring the empty vector pHERD20T or an arabinose-inducible *amrZ* construct cloned into pHERD20T were grown in biofilm tube reactors in 5-fold diluted VBMM. The growth medium was supplemented with 8 μg/mL carbenicillin for the plasmid maintenance of pHERD20T. After 5 days of growth, arabinose was added to the growth medium at a concentration of 1% to induce the expression of *amrZ*. Effluent from the biofilms was collected for 90 min at 1 min intervals, and the absorbance was subsequently recorded at 600 nm. (B) Dispersion response of 5-day-old biofilms by PAO1 harboring an arabinose-inducible *bdlA*_G31A construct cloned into pJN05 after the addition of 1% arabinose. Differently colored lines represent individual dispersion responses from at least 3 biological replicates.

Considering that the lack of *amrZ* expression rendered biofilms by P. aeruginosa dispersion-deficient in response to glutamate and nitric oxide, we next asked whether the overexpression of *amrZ* leads to dispersion. We anticipated that these conditions would be conducive to dispersion, as AmrZ has been reported to activate the expression of *endA* and *pelA* (67), two genes which have been previously reported to induce dispersion (40, 41). Therefore, we made use of strain dt*amrZ*/pHERD*-amrZ*, which allows for the arabinose-induced expression of *amrZ*. Biofilms by dt*amrZ*/pHERD*-amrZ* were grown for 5 days in biofilm tube reactors, and dispersion was subsequently induced by exposing the biofilms to 1% arabinose to induce *amrZ* gene expression. Biofilms by dt*amrZ*/pHERD harboring the empty vector pHERD20T were used as a control and failed to disperse in response to arabinose (Fig. 2). Likewise, biofilms by dt*amrZ*/pHERD*-amrZ* did not disperse upon a challenge with arabinose (Fig. 2). This is in contrast to biofilms by PAO1/pJN-bdlA_G31A, which dispersed under the conditions tested (Fig. 2).

**FIG 3:**
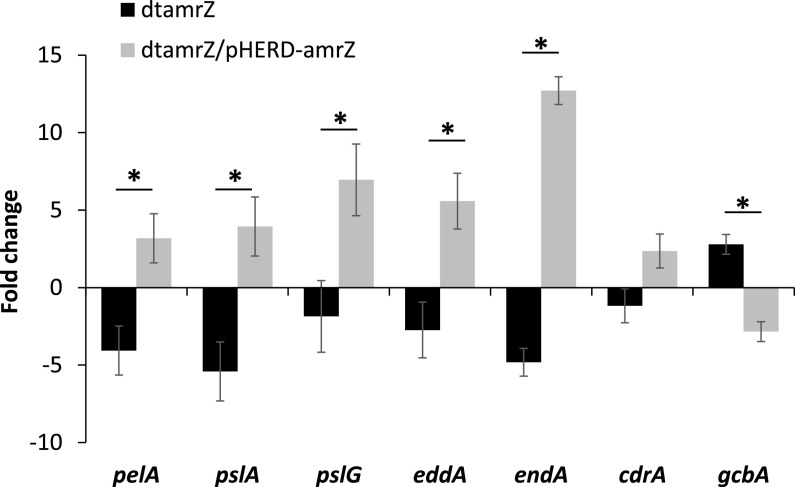
Dependency of several known or hypothetical matrix-degrading enzymes and matrix components on AmrZ. qRT-PCR experiments were performed on 3-day-old biofilm cells grown in biofilm tube reactors with 5-fold diluted VBMM. The transcript abundance values of genes obtained from dt*amrZ* mutant biofilms were compared to those of the wild-type for reference, whereas the plasmid-complemented strain (dt*amrZ*/pHERD-*amrZ*) was compared to an empty vector control (dt*amrZ*/pHERD20T). Plasmid-complemented and empty vector strains were grown in the presence of 0.1% arabinose and 8 μg/mL carbenicillin for plasmid maintenance. *cysD* was used as the housekeeping gene. Statistical analysis was performed using a two-tailed *t* test (*, *P* < 0.05). Error bars represent the standard deviation.

Given the somewhat surprising result of the induction of *amrZ* overexpression not resulting in dispersion, we asked whether, under the conditions tested, the plasmid-borne expression of *amrZ* leads to the elevated expression of matrix-degrading components in 3-day-old biofilms, using qRT-PCR. The genes of interest included *pelA*, *pslG*, *eddA*, and *endA* (40, 41, 45). In addition, the transcript abundance of *cdrA* encoding the CdrA adhesin (40, 75, 76) and *gcbA* (PA4843) encoding a diguanylate cyclase GcbA (13, 67, 77) were evaluated. Biofilms formed by PAO1 and dt*amrZ*/pHERD were used as controls. Relative to those of the wild-type biofilms, the transcript abundance values of *pslG*, *pelA*, *eddA*, and *endA* were significantly decreased in the biofilms formed by dt*amrZ*/pHERD but were significantly increased in the dt*amrZ*/pHERD*-amrZ* biofilms (Fig. 3). The results are in agreement with those of previous reports (67), further confirming that under the conditions tested, AmrZ contributes to the transcript abundance of matrix degrading factors. However, *cdrA* was significantly increased in biofilms overexpressing *amrZ*. In contrast, *gcbA* was significantly decreased (Fig. 3), highlighting the findings of AmrZ repressing *gcbA* but increasing *cdrA* transcript abundance, conditions which have previously been shown to impede dispersion (13, 35, 40, 78).

### Dispersion induced by PelA- and EndA-dependent matrix degradation is dependent on AmrZ.

**FIG 4:**
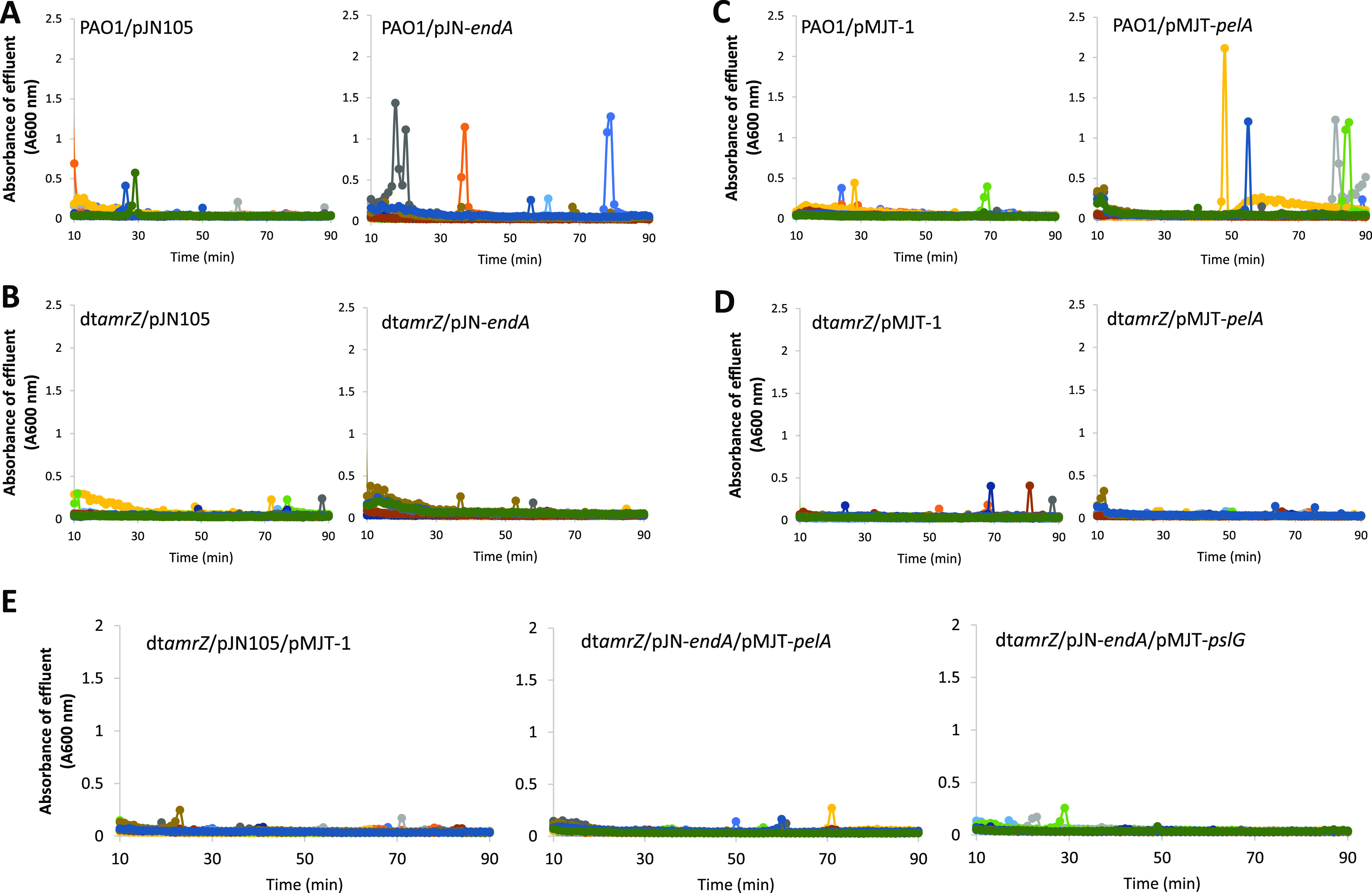
Overexpression of genes encoding matrix-degrading enzymes does not restore the dispersion response by dt*amrZ* biofilms. Biofilms were grown for 5 days in biofilm tube reactors with 5-fold diluted VBMM prior to the induction of gene expression. The growth medium was supplemented with 8 μg/mL carbenicillin for maintenance of the pMJT-1 plasmid and 2 μg/mL gentamicin for the pJN105 plasmid. The expression of genes of interest was induced by the addition of 1% arabinose to the growth medium. Effluent from the biofilms was collected for 90 min at 1 min intervals and the absorbance was subsequently recorded at 600 nm. Response of biofilms formed by (A) PAO1 and (B) dt*amrZ* harboring the empty vector pJN105 or expressing *endA* in response to the arabinose-induced gene expression of *endA*. Response of biofilms formed by (C) PAO1 and (D) dt*amrZ* harboring the empty vector pMJT-1 or expressing *pelA* in response to the arabinose-induced gene expression of *pelA*. Response of biofilms formed by (E) dt*amrZ* harboring the empty vectors pMJT-1 and pJN105 or coexpressing *pelA* and *endA* or *pslG* and *endA* after addition to arabinose to induce gene expression. Differently colored lines represent individual dispersion responses from at least 3 biological replicates.

Our findings confirmed that AmrZ contributes to the expression of matrix-degrading factors *pslG*, *pelA*, *eddA*, and *endA.* We previously demonstrated that the overexpression of genes encoding matrix degrading enzymes, specifically hydrolase PelA and endonuclease EndA, was sufficient to induce dispersion by P. aeruginosa biofilms (40, 41). To determine whether this response requires the presence of AmrZ, we constructed dt*amrZ* strains that were overexpressing *pelA* or *endA* under the control of a p_BAD_ promoter. The respective biofilms were grown for 5 days in biofilm tube reactors, and dispersion was subsequently induced by exposing the biofilms to arabinose to induce the expression of *pelA* or *endA*. In agreement with previous findings (40, 41), wild-type biofilms overexpressing *pelA* and *endA* (PAO1/pJN-*endA*, PAO1/pMJT-*pelA*) dispersed under the conditions tested (Fig. 4A and C), whereas the PAO1 biofilms harboring empty vectors (pJN105, pMJT-1) failed to disperse upon a challenge with arabinose (Fig. 4A and C). In contrast, dt*amrZ* biofilms overexpressing *pelA* or *endA* failed to disperse (Fig. 4B and D). Our findings strongly suggest that the dispersion induced by matrix degradation via PelA and EndA is dependent on AmrZ.

### Dispersion induced by PslG-dependent matrix degradation is dependent on AmrZ.

**FIG 5:**
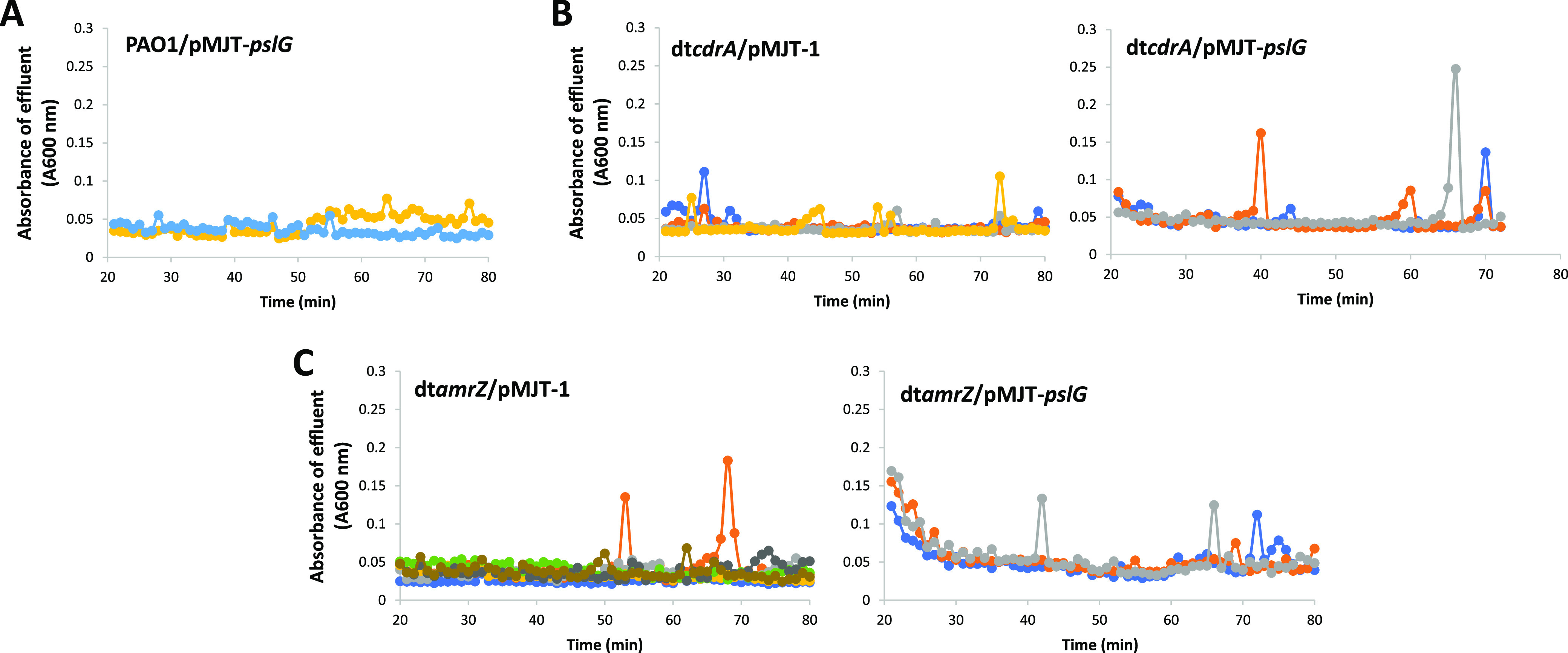
Overexpression of *pslG* dispersed biofilms formed by dt*cdrA* does not restore the dispersion response by dt*amrZ* biofilms. Biofilms were grown for 5 days in in biofilm tube reactors with 5-fold diluted VBMM prior to the induction of gene expression. The growth medium was supplemented with 8 μg/mL carbenicillin for the maintenance of the pMJT-1 plasmid. Expression of *pslG* was induced by the addition of 1% arabinose to the growth medium. Effluent from the biofilms was collected for 90 min at 1 min intervals, and the absorbance was subsequently recorded at 600 nm. (A) Dispersion profile of biofilms by PAO1 in response to the arabinose-induced gene expression of *pslG.* (B) Dispersion profile of biofilms by dt*cdrA* vector control and dt*cdrA*/pMJT-*pslG* in response to the arabinose-induced gene expression of *pslG.* (C) Dispersion profile of biofilms by dt*amrZ* vector control and dt*amrZ*/pMJT-*pslG* in response to the arabinose-induced gene expression of *pslG.* Differently colored lines represent individual dispersion responses from at least 3 biological replicates.

Previous findings indicated that the overexpression of *pslG* encoding a Psl polysaccharide hydrolase coincided with dispersion; however, dispersion was only noted in strains lacking the matrix adhesin CdrA (40). Considering that the inactivation of *amrZ* coincides with reduced *cdrA* transcript abundance (67), we next explored the role of *pslG* in dispersion. In agreement with previous findings, no dispersion events were detected for biofilms by PAO1/pMJT-*pslG* (Fig. 5A). While biofilms by PAO1/pMJT-*pslG* failed to disperse, the induction of *pslG* gene expression in a *cdrA* mutant background coincided with the dispersion, apparent by sharp increases in the absorbance values (600 nm) of the effluent, which are indicative of dispersion events (27, 41) (Fig. 5B). However, despite a reduced *crdA* transcript abundance, biofilms by dt*amrZ*/pMJT-*pslG* did not disperse in a manner comparable to that of biofilms by dt*cdrA*/pMJT-*pslG*. (Fig. 5B and C). This was apparent by the much-reduced dispersion events displayed by biofilms formed by dt*amrZ*/pMJT-*pslG*, relative to those displayed by biofilms formed by dt*cdrA*/pMJT-*pslG*, with the extent of the dispersion events being comparable to those displayed by the vector control strain dt*amrZ*/pMJT-1 (Fig. 5B and C). Our findings strongly suggest that dispersion induced by matrix degradation via PslG requires the absence of CdrA to ensure the untethering of the Psl polysaccharide (40) and that it depends on AmrZ for additional regulation.

Considering that AmrZ contributes to the abundance of more than one matrix-degrading enzyme (67), we next asked whether more than one matrix-degrading enzyme is required to restore the dispersion response by dt*amrZ* biofilms. Therefore, we determined whether overproducing the two matrix-degrading enzymes, PelA and EndA, would enable dispersion by evaluating the dispersion responses of biofilms formed by dt*amrZ*/pJN-*endA*/pMJT-*pelA.* However, similar to the vector control dt*amrZ*/pJN105/pMJT-1, the mutant biofilms failed to disperse upon the induction of gene expression by arabinose (Fig. 4E). Similar results were obtained for the biofilms formed by dt*amrZ*/pJN-*endA*/pMJT-*pslG* (Fig. 4E).

### AmrZ works in concert with BdlA to enable dispersion.

The chemotaxis transducer protein BdlA is central to the dispersion response (37). This is supported by Δ*bdlA* biofilms being impaired in the dispersion response to various dispersion cues, including heavy metals, glutamate, and nitric oxide (11, 12). However, for BdlA to contribute to dispersion, the protein first needs to be activated via a process requiring elevated c-di-GMP levels, BdlA phosphorylation, and the nonprocessive proteolytic cleavage of BdlA (12, 27). The BdlA variant BdlA_G31A mimics activated BdlA, transmitting a constant signal-on bias for dispersion (27). Therefore, biofilms overexpressing *bdlA*_G31A are hyperdispersive (27), apparent by their reduced biofilm biomass accumulation and a 2 to 3-fold increase of bacteria present in biofilm effluents, compared to wild-type biofilms over the course of 5 days of biofilm growth (27), as well as by a significant increase in the transcript abundance of genes encoding DNA endonucleases (*endA*, *eddA*) and hydrolases (*pelA*, *pslG*) (40, 41).

**FIG 6:**
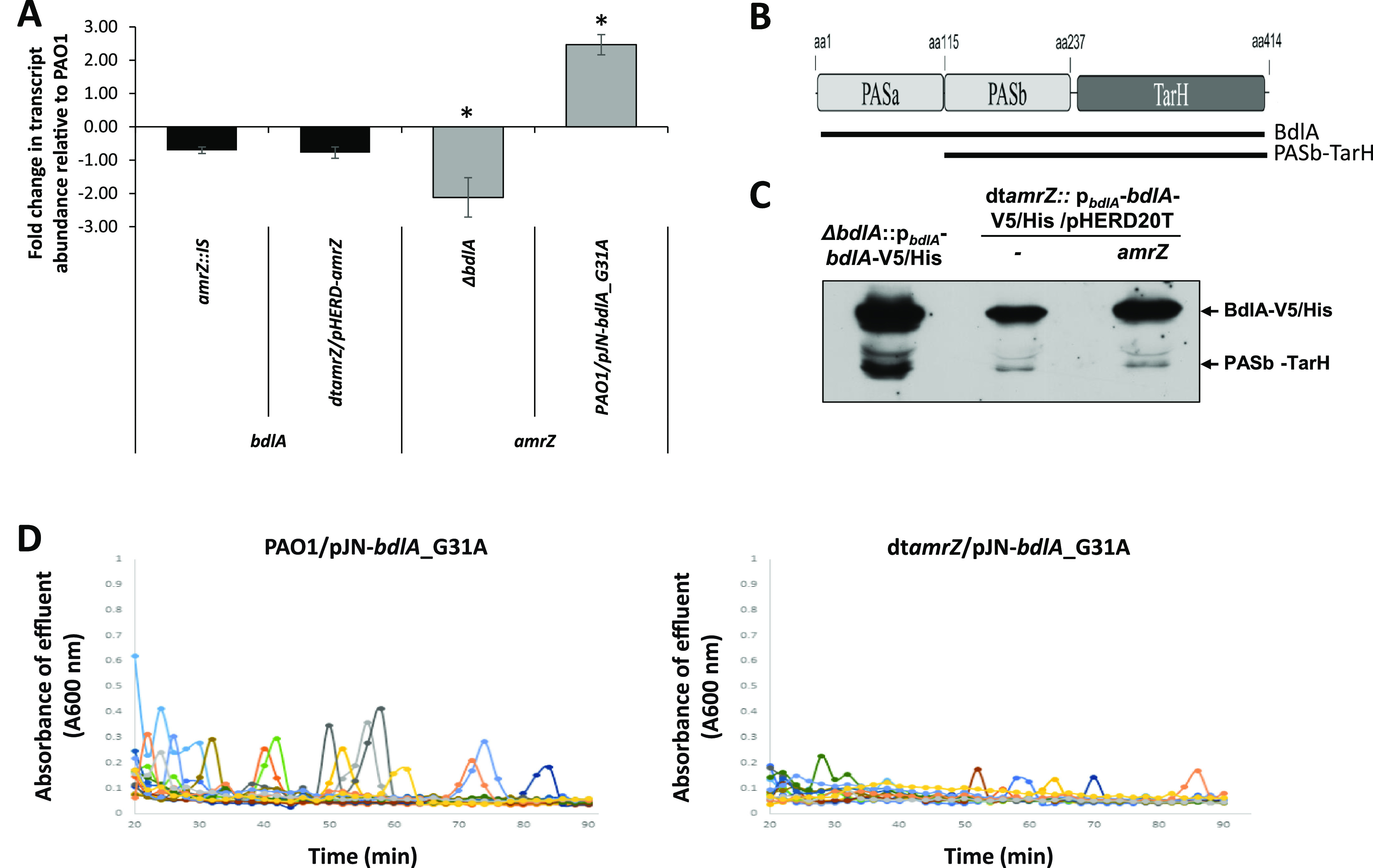
The hyperdispersive phenotype of PAO1/pJN-*bdlA*_G31A is dependent on AmrZ. (A) Transcript abundance of *bdlA* and *amrZ*, as determined by qRT-PCR. RNA isolated from 5-day-old biofilms formed by the indicated strains were used, and the fold change in transcript abundance was determined relative to the transcript abundance values of wild-type biofilms. *cysD* was used as the housekeeping gene. The experiments were done in triplicate, and the standard deviation is shown. An asterisk denotes a statistically significant difference (*P* < 0.05) relative to the PAO1 control strain, as determined using a one-way ANOVA, followed by Dunnett’s *post hoc* test. (B) The domain structure of BdlA. Cleavage occurs between the two PAS domains, resulting in PASa and PASb-TarH. Using C-terminally tagged BdlA, only intact BdlA and the cleaved PASb-TarH fraction are detectable via an immunoblot analysis using anti-V5 antibodies. (C) Image of an immunoblot showing the abundance of intact BdlA and cleaved C-terminally tagged BdlA_V5/His present in total cell extracts obtained from 3-day-old biofilms by *ΔbdlA*, dt*amrZ*, and dt*amrZ*/pHERD-*amrZ*. The respective strains harbor a chromosomally inserted, C-terminally tagged BdlA_V5/His under the control of its own promoter. The biofilms were grown in 5-fold diluted VBMM. Intact and cleaved BdlA was detected using anti-V5 antibodies. The experiments were carried out in triplicate, and a representative image is shown. (D) Biofilms formed by PAO1 and dt*amrZ* harboring an arabinose-inducible *bdlA*_G31A construct cloned into pJN105 were grown as biofilms in tube reactors in 5-fold diluted VBMM. The growth medium was supplemented with 2 μg/mL gentamicin for the plasmid maintenance of pJN105. After 5 days of growth, arabinose was added to the growth medium at a concentration of 1% to induce the expression of *bdlA*_G31A. Effluent from the biofilms was collected for 90 min at 1 min intervals, and the absorbance was subsequently recorded at 600 nm. The individual dispersion responses from at least 3 biological replicates are indicated by colored lines. Each biological replicate consisted of 4 technical replicates.

Given the similarity of the genes affected by BdlA_G31A and AmrZ, we asked whether the transcript abundance of *bdlA* is dependent on AmrZ. To do so, we evaluated the transcript abundance of *bdlA* in the absence and presence of *amrZ* by qPCR. As shown in Fig. 6A, no difference in the *bdlA* transcript abundance was noted when biofilms by dt*amrZ/*pHERD20T and dt*amrZ/*pHERD*-amrZ* were compared. In contrast, significant differences in *amrZ* transcript abundance were noted in the absence and presence of *bdlA* (Fig. 6A), supporting the notion that *bdlA* expression is not dependent on AmrZ.

Considering that AmrZ does not affect the transcript abundance of *bdlA*, we next explored whether the absence or presence of AmrZ affected the activation of BdlA. BdlA activation is apparent by the cleavage of BdlA at position methionine-130 (M130), which is located between the two BdlA-PAS domains, PASa and PASb, resulting in two fragments composed of PASa and PASb-TarH (12) (Fig. 6B). Therefore, we made use of immunoblot analysis to detect intact and cleaved BdlA in biofilms formed by dt*amrZ* strains harboring the empty vector pHERD20T or overexpressing *amrZ* under the control of a P*_BAD_* promoter. The strains harbored a chromosomally located, C-terminally tagged *bdlA*_V5/His under the control of its own promoter. No difference in BdlA processing was noted between dt*amrZ/*pHERD20T and dt*amrZ/*pHERD*-amrZ*, apparent by the presence of both C-terminally tagged intact BdlA and the BdlA cleavage product, PASb-TarH (Fig. 6C). The same protein bands were detectable in biofilms by *ΔbdlA*::P*_bdlA_*-*bdlA*-V5/His, which were used as positive-control (12) (Fig. 6C). The findings indicated that AmrZ did not affect BdlA activation.

Instead of *bdlA* expression or BdlA activation being dependent on AmrZ, our findings suggested that AmrZ functions downstream of BdlA, apparent by BdlA affecting the transcript abundance of *amrZ* (Fig. 6A) with the overexpression of *bdlA*_G31A, thus creating dispersive biofilm conditions (27) and coinciding with increased *amrZ* expression (Fig. 6A). To further explore the functional relationship between BdlA and AmrZ, we next asked whether AmrZ indeed functions downstream of constitutively active BdlA. We reasoned that if BdlA functionality requires AmrZ, then overexpressing *bdlA*_G31A in a dt*amrZ* mutant background would not result in hyperdispersive conditions. As anticipated, the induction of *bdlA*_G31A expression in a dt*amrZ* mutant background resulted in few to no detectable dispersion events (Fig. 6D). In contrast, and in agreement with previous findings (27, 41), PAO1 biofilms overproducing BdlA_G31A dispersed (Fig. 6D). The findings indicated that dispersion via BdlA requires AmrZ, with AmrZ likely functioning downstream of BdlA.

### Identification of novel factors contributing to dispersion.

Our findings so far suggest that AmrZ functions in concert with (albeit downstream of) BdlA and that matrix degradation that leads to dispersion is likely AmrZ-dependent. However, our data also suggested that dispersion requires factors in addition to matrix degradation. We reasoned that such factors are AmrZ-dependent and are induced upon the induction of dispersion. To identify such factors, we first screened an RNA-seq data set that was published by Jones et al. (67) for AmrZ-induced genes and limited the selection to genes encoding hypothetical proteins and proteins not previously linked to dispersion. By doing so, we selected 8 genes (Table 1). These included *napB*, which encodes a cytochrome c type protein (with homologs previously linked to nitrogen metabolism in E. coli [79]), *vreA*, and *vreR*, which are involved in the regulation of cell surface signaling and virulence (80, 81), and PA2933, which encodes an efflux protein of the major facilitator superfamily that has been previously linked to autoaggregation and to the formation of wrinkled colonies (82). The remaining genes comprised PA2655, PA2750, PA2819, and PA1891, which encode uncharacterized hypothetical proteins with unknown functions (83). We then asked whether the respective genes were induced upon dispersion. As biofilms inactivated in or overexpressing *amrZ* are nondispersive (Fig. 1 and 2), we mimicked dispersion-inducing conditions by overexpressing *bdlA*_G31A. We reasoned that genes that are increased under dispersion-mimicking conditions in the wild-type biofilms but not in the dt*amrZ* mutant biofilms likely contribute to the dispersion response in a manner that is dependent on AmrZ.

**TABLE 1:** Fold change in the transcript abundance of 8 potential AmrZ-target genes under nondispersive and hyperdispersive conditions^*c*^

		Fold change^*a*^		
Gene	Description	RNA-seq^*b*^ (PAO1 relative to dt*amrZ*)	dt*amrZ*/pJN-*bdlA*_G31A relative to dt*amrZ*	PAO1/pJN-bdlA_G31A relative to dt*amrZ*
*napB*	Energy metabolism	2.36	−2.04 ± 0.25*	3.56 ± 0.56*
PA2655	Hypothetical, unclassified, unknown	3.14	1.13 ± 0.73	1.52 ± 1.01
PA2750	Hypothetical, unclassified, unknown	5.15	−1.21 ± 0.13	1.11 ± 0.29
PA2819	Hypothetical, unclassified, unknown	2.48	1.04 ± 0.32	1.15 ± 0.26
PA1891	Membrane proteins	2.25	2.14 ± 0.51*	1.95 ± 0.37*
PA2933	Membrane proteins, transport of small molecules	2.03	−1.00 ± 0.84	1.82 ± 2.02
*vreR*	Protein secretion/export apparatus	2.29	1.25 ± 0.24	1.32 ± 0.41
*vreA*	Transcriptional regulators	2.67	1.02 ± 0.32	−1.08 ± 0.28

a Data taken from Jones et al. (67).

b A positive number indicates a greater expression in biofilms formed by PAO1, PAO1/pJN-*bdlA*_G31A, or dt*amrZ*/pJN-*bdlA*_G31A relative to dt*amrZ*.

c Strains tested include the hyperdispersive PAO1/pJN-*bdlA*_G31A, the nondispersive dt*amrZ*::Tet/pJN-*bdlA*_G31A, and dt*amrZ*, which was used as the reference strain. qRT-PCR was used to determine the transcript abundance of indicated genes and was performed using 3-day-old biofilms grown in biofilm tube reactors in 5-fold diluted VBMM. Strains harboring the pJN105 plasmid were grown in the presence of 0.1% arabinose to induce the expression of *bdlA*_G31A and 8 μg/mL carbenicillin for plasmid maintenance. Relative transcript abundance was determined for 8 genes that were selected from the 16 potential AmrZ target genes. Fold changes in the transcript abundance of >2 or <−2 were considered to be the minimum thresholds for biological significance. *cysD* was used as the housekeeping gene. Statistical analysis was conducted using a one-way ANOVA for each tested gene, followed by Dunnett’s *post hoc* test, to detect statistically significant differences between strains. An asterisk denotes a statistically significant difference (*P* < 0.05), relative to the dt*amrZ* control strain. The fold changes exhibited by these 8 genes in the RNAseq data sets and descriptions of their function are taken from Jones et al. (67). The experiments were carried out in triplicate. The ± symbol indicates the standard deviation.

Under the conditions tested, no significant (>2 or <−2-fold) difference in transcript abundance was noted for PA2655, PA2750, PA2819, *vreA*, or *vreR* (Table 1). PA2933 was significantly reduced in biofilms by PAO1/pHERD-*bdlA*_G31A, relative to the vector control strain (Table 1). Therefore, the respective genes (PA2933, PA2819, PA2750, PA2655, *vreA*, *vreR*) were excluded from further analysis. PA1891 was found to be significantly increased in transcript abundance upon the induction of *bdlA*_G31A in biofilms by both PAO1 and dt*amrZ* (Table 1), suggesting that the presence or absence of AmrZ does not affect PA1891 expression under *bdlA*_G31A-induced dispersion conditions. The only gene demonstrating AmrZ-dependency was *napB*, with the transcript abundance of *napB* being increased in hyperdispersive cells (PAO1/pHERD-*bdlA*_G31A) and reduced in nondispersive biofilms formed by dt*amrZ*/pHERD-*bdlA*_G31A (Table 1).

### Insertional inactivation of *napB* or PA1891 impairs biofilm dispersion in response to nitric oxide.

As indicated above, we assumed that genes that are increased upon the overexpression of *bdlA*_G31A in the wild-type but not in the dt*amrZ* mutant biofilms likely contribute to the dispersion response in a manner that is dependent on AmrZ. Among the 8 genes tested, the transcript abundance of only *napB* increased in hyperdispersive cells and decreased in nondispersive biofilms. In contrast, the transcript abundance of PA1891 was increased upon the expression of *bdlA*_G31A, regardless of the dispersion phenotype.

**FIG 7:**
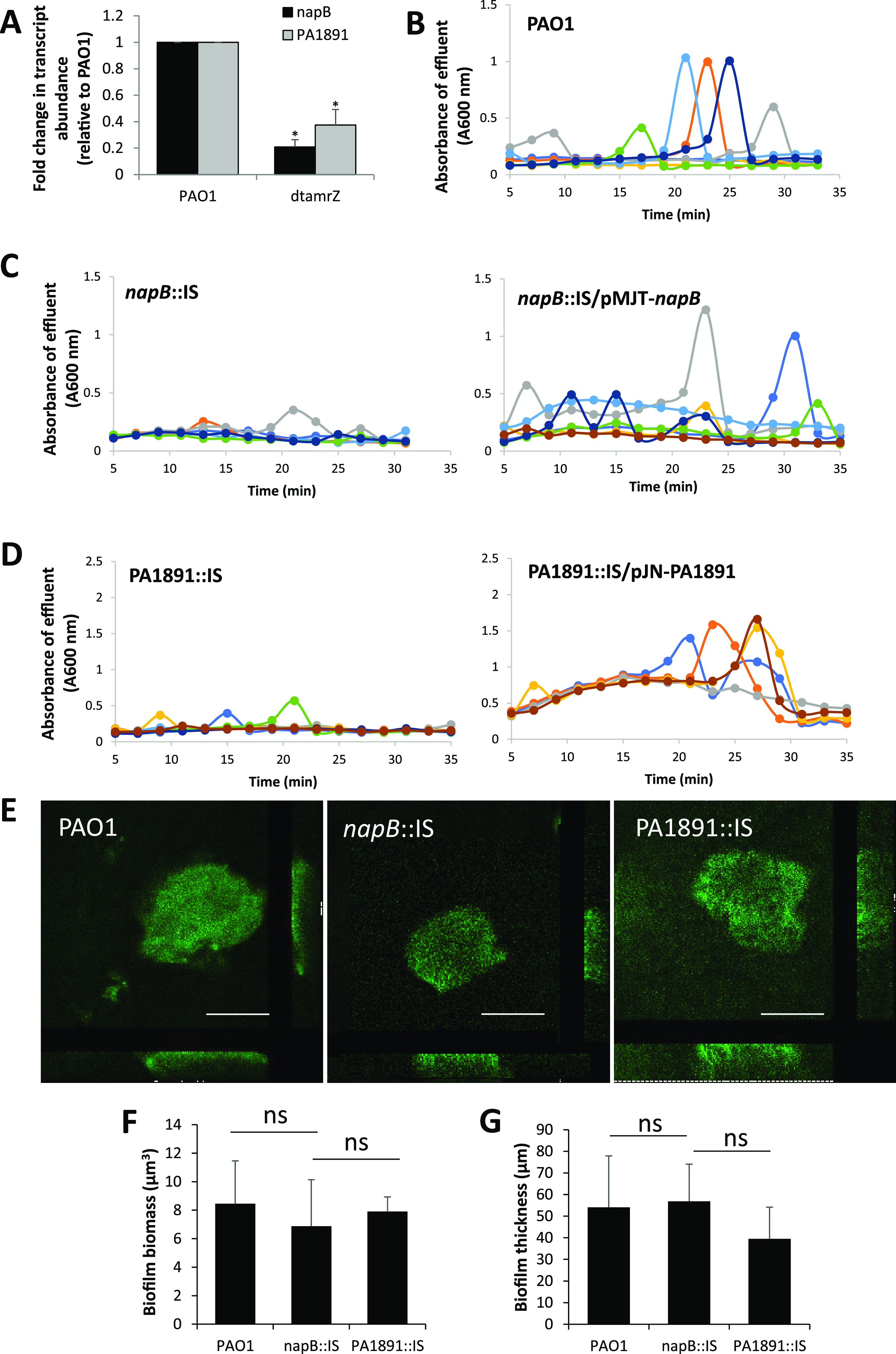
Contribution of AmrZ-targets *napB* and PA1891 in the dispersion response. Biofilms by PAO1, *napB*::IS, and PA1891::IS were grown in 5-fold diluted VBMM in continuous flow biofilm tube reactors. (A) qRT-PCR experiments were performed on 5-day-old biofilm cells grown in biofilm tube reactors with 5-fold diluted VBMM. The transcript abundance values of *napB* and PA1891 obtained from dt*amrZ* mutant biofilms were compared to those of the wild-type for reference. *cysD* was used as the housekeeping gene. An asterisk indicates a statistically significantly difference from PAO1 (*P* < 0.05), as determined using a one-way ANOVA, followed by Dunnett’s *post hoc* test. (B–D) Dispersion was induced after 5 days of growth via the addition of sodium nitroprusside as a source of nitric oxide. Effluents from tube reactors of biofilms by (B) P. aeruginosa PAO1, (C) *napB*::IS and napB::IS/pMJT-*napB*, and (D) PA1891::IS and the same strain overexpressing PA1891 were collected for 35 min at 1 min intervals. The absorbance was determined by spectrophotometry at 600 nm. Colored lines represent individual dispersion responses from at least 3 biological replicates. (E) Representative confocal images of biofilms formed by the indicated strains grown for 5 days under continuous flow conditions. Scale bar, 100 μm. Biofilm biomass (F) and biofilm thickness (G) by PAO1 and the respective mutant strains was determined using COMSTAT analysis. ns, not significant, as determined using an ANOVA, followed by Dunnett’s *post hoc* test.

To ensure the AmrZ-dependency of PA1891 and *napB* under the conditions tested, we evaluated the transcript abundance in 5-day-old biofilms formed by PAO1 and dt*amrZ* via qRT-PCR. In agreement with previous findings (67), the transcript abundance of PA1891 and *napB* was significantly reduced in biofilms by dt*amrZ*, relative to those of the wild-type biofilms (Fig. 7A).

We next examined the role of *napB* and PA1891 in the dispersion response. To do so, we used a mutant strain harboring a transposon insertion in *napB*, referred to as *napB*::IS, and PA1891, referred to as PA1891::IS. Biofilms formed by the respective mutant strains were grown for 5 days under flowing conditions. After 5 days of growth, the biofilms were subsequently exposed to SNP as a source of nitric oxide to induce dispersion. Biofilms by PAO1 were used as controls. Exposure of the wild-type biofilms to nitric oxide coincided with a sharp increase in the absorbance of the biofilm effluent (Fig. 7B), a response that was absent in the biofilms formed by *napB*::IS (Fig. 7C). Similar to the *napB*::IS biofilms, biofilms formed by PA1891::IS failed to disperse in response to nitric oxide (Fig. 7D). To exclude polar effects of the transposon insertion in *napB*::IS and PA1891::IS, we determined whether complementation restored the dispersion response. The multicopy expression of *napB* or PA1891 in the respective mutant strains restored dispersion by *napB*::IS or PA1891::IS biofilms in response to nitric oxide to the levels displayed by the wild-type (Fig. 7C and D).

To ensure that the lack of dispersion by *napB*::IS and PA1891::IS mutant strains is not due to a lack of biofilm formation, we quantitatively analyzed the biofilm architectures of the two mutant strains via confocal microscopy and, subsequently, via COMSTAT. Under the conditions tested, *napB*::IS and PA1891::IS formed structured biofilms that were comparable to those of the PAO1 biofilms (Fig. 7E–G).

### PA1891 is required for BdlA to induce dispersion.

**FIG 8:**
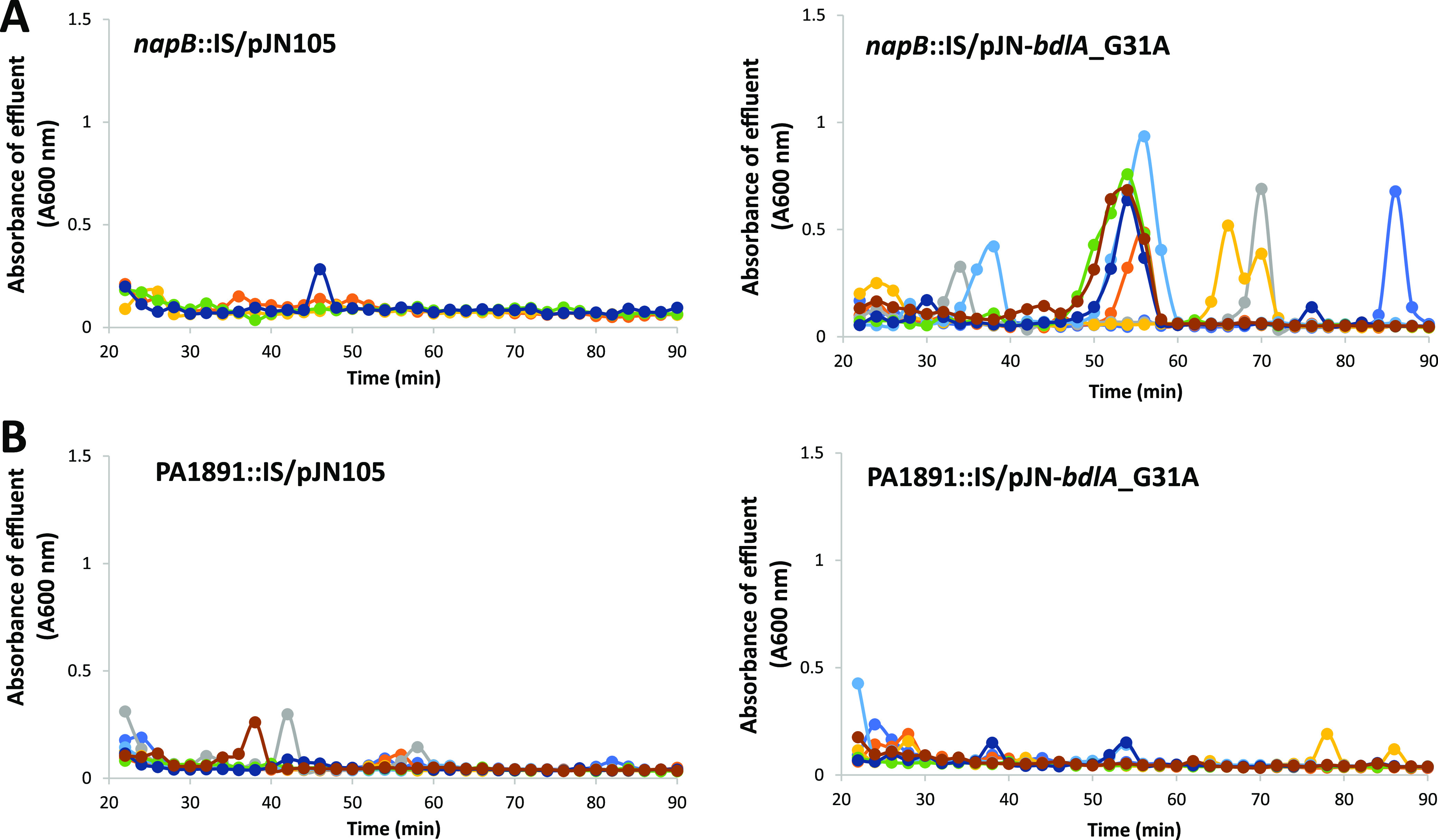
The hyperdispersive response of biofilms overexpressing *bdlA*_G31A is dependent on PA1891 but not *napB*. Biofilms by *napB*::*IS*, PA1891::IS, and the respective mutant strains harboring an arabinose-inducible *bdlA*_G31A construct cloned into pJN05 were grown as biofilms in tube reactors in 5-fold diluted VBMM with 2 μg/mL gentamicin for plasmid maintenance. After 5 days of growth, 1% arabinose was added to the growth medium to induce the expression of *bdlA*_G31A. The effluents from the tube reactors were collected for 90 min, and the absorbance was determined by spectrophotometry at 600 nm. (A) Absorbance of effluents by biofilms formed by *napB*::IS/pJN105 and *napB*::IS/pJN-*bdlA*_G31A after the addition of arabinose. (B) Absorbance of effluents by biofilms formed by PA1891::IS/pJN105 and PA1891::IS/pJN-*bdlA*_G31A after the addition of arabinose. The colored lines represent individual dispersion responses from at least 3 biological replicates, each of which consisted of 2 to 4 technical replicates.

To determine whether the *napB*::IS biofilm is defective in dispersion cue perception but otherwise retains its capability to disperse, we next determined the dispersion response by this mutant strain following the induction of *bdlA*_G31A expression. To accomplish this, *napB*::IS/pJN-*bdlA*_G31A and the respective control strain were grown for 5 days, at which time dispersion was induced by the addition of 1% arabinose. Under the conditions tested, the effluents of biofilms by *napB*::IS/pJN-*bdlA*_G31A demonstrated sharp increases in turbidity, and these increases were absent in the control strain (Fig. 8A). The findings suggested that while *napB* is expressed in an AmrZ- and dispersion-dependent manner and is required for dispersion in response to nitric oxide, the strain retains its dispersion capability.

Given that the transcript abundance of PA1891 was increased upon the expression of *bdlA*_G31A, regardless of the dispersion phenotype, we hypothesized that PA1891 was required for dispersion, irrespective of the conditions tested. Therefore, we asked whether the induction of *bdlA*_G31A expression rendered biofilms formed by PA1891::IS dispersive or not. Under the conditions tested, no differences were noted between the effluents of biofilms by PA1891::IS/pJN-*bdlA*_G31A and the control strain PA1891::IS (Fig. 8B). The findings strongly suggested that PA1891 was essential for dispersion in response to nitric oxide and under hyperdispersive conditions and was initiated by the overexpression of *bdlA*_G31A, by P. aeruginosa biofilms.

### Expression of *napB* and PA1891 restores the dispersion phenotype by the dt*amrZ* mutant.

We next explored the question of whether *napB* and PA1891 are responsible for the impaired dispersion response by dt*amrZ* biofilms. Therefore, we asked whether expressing *napB* or PA1891 from an inducible promoter restores the dispersion response by the dt*amrZ* mutant.

**FIG 9:**
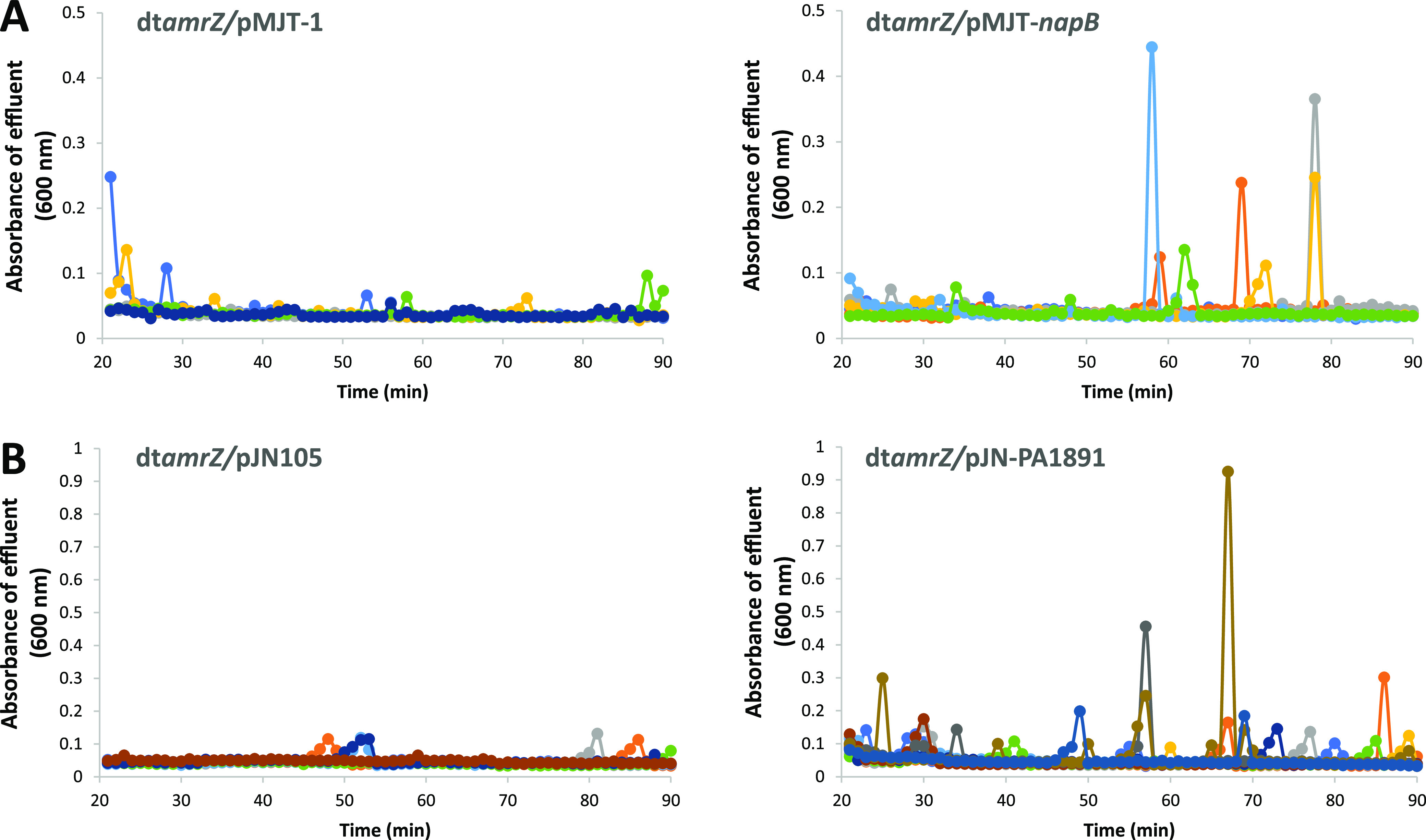
Multicopy expression of *napB* and PA1891 restore the dispersion response by dt*amrZ* biofilms. (A) Biofilms by dt*amrZ* harboring an arabinose-inducible *napB* construct cloned into pMJT-1 were grown in tube reactors in 5-fold diluted VBMM with 8 μg/mL carbenicillin for plasmid maintenance. After 5 days of growth, 1% arabinose was added to the growth medium to induce the expression of *napB*. After the induction of gene expression, biofilm effluents were collected for 90 min, and the absorbance was determined by spectrophotometry at 600 nm. Biofilms by dt*amrZ* harboring the empty plasmid pMJT-1 were used as controls. (B) Biofilms by dt*amrZ* harboring an arabinose-inducible PA1891 construct cloned into pJN105 were grown as biofilms in tube reactors in 5-fold diluted VBMM with 2 μg/mL gentamicin for plasmid maintenance. After 5 days of growth, 1% arabinose was added to the growth medium to induce the expression of PA1891, and biofilm effluents were subsequently collected for 90 min. The absorbance of biofilm effluents was determined by spectrophotometry at 600 nm. Biofilms by dt*amrZ* harboring an empty vector were used as controls. The colored lines represent individual dispersion responses from at least 3 biological replicates, each of which consisted of 2 to 4 technical replicates.

Biofilms by dt*amrZ* that are expressing *napB* or PA1891 from a plasmid under the control of an arabinose inducible promoter were grown in biofilm in tube reactors under flowing conditions for 5 days, and dispersion was subsequently induced by exposing the biofilms to 1% arabinose to induce *napB* or PA1891 gene expression. dt*amrZ* harboring empty vectors (pJN105, pMJT-1) were used as controls. As anticipated, biofilms formed by dt*amrZ*/pMJT-1 failed to disperse upon the addition of arabinose (Fig. 9A). In contrast, biofilms by dt*amrZ*/pMJT-*napB* dispersed following the induction of *napB* expression (Fig. 9A). Likewise, biofilms by dt*amrZ*/pJN-PA1891 dispersed following the induction of PA1891 expression, whereas dt*amrZ* harboring the empty plasmid pJN105 did not (Fig. 9B). It is of interest to note that the induction of gene expression of *napB* and, in particular, PA1891, coincided with multiple dispersion events throughout the experiment (Fig. 9), likely suggesting that the respective biofilms are hyperdispersive. Overall, our findings strongly suggested that *napB* and PA1891 contribute to the impaired dispersion response by dt*amrZ* biofilms.

## DISCUSSION

Prior research has focused on dispersion cue perception, the relay of dispersion cue sensing, and biofilm matrix degradation to enable the release of cells from the biofilm matrix. However, events leading to matrix degradation upon dispersion cue sensing, resulting in an overall reduction of the biofilm population and leading to dispersion, have remained elusive. While the intracellular signaling molecule c-di-GMP has been linked to many of the phenotypic changes ascribed to dispersion, including the modulation of motility and matrix production, little is known about the regulatory mechanisms leading to matrix degradation and to cells actively leaving the biofilm.

Here, we report that the alginate and motility regulator AmrZ plays an essential role in the dispersion response, linking dispersion cue sensing via BdlA to matrix degradation and, ultimately, to the liberation of bacterial cells from the biofilm. This is supported by dt*amrZ* biofilms being nondispersive in response to nitric oxide and glutamate (Fig. 1), failing to disperse upon the induction of *bdlA*_G31A gene expression (Fig. 2), and likely functioning downstream of BdlA, after dispersion cue sensing (Fig. 6B–D). Moreover, the gene *amrZ* was found to be significantly upregulated in biofilm cells after the induction of *bdlA*_G31A gene expression, a condition that simulates dispersion (Fig. 6A).

The finding of AmrZ contributing to dispersion is in agreement with the notion that AmrZ modulates the expression levels of several known and hypothetical matrix hydrolases and nucleases (67), some of which (*endA*, *pelA*, and *pslG*) have been identified as active factors in biofilm dispersal (40, 41). Both the *pel* and *psl* operons are directly regulated by AmrZ, and *endA* expression shows a strong correlation with AmrZ, but its promoter does not contain AmrZ binding sites (67). In agreement with previous studies, we demonstrated here that the induction of *endA*, *pelA*, and/or *pslG* results in dispersion by P. aeruginosa biofilms. However, biofilms by dt*amrZ* did not disperse upon the induction of *endA* and/or *pelA*. Likewise, P. aeruginosa biofilms overexpressing *amrZ* failed to disperse. However, we confirmed that the lack of dispersion was not due to the insufficient expression of *endA*, *pelA*, or *pslG* (via qRT-PCR) (67) (Fig. 3), or to the overexpression of *endA*, *pslG*, and/or *pelA* in biofilms formed by dt*amrZ* (Fig. 4 and 5). Instead, it seems as though dispersion by biofilms formed by dt*amrZ* requires either (i) more than eDNA and one of the polysaccharides (Pel or Psl) to be degraded (Fig. 4 and 5), (ii) additional factors to enable the dispersion response, or (iii) factors to enable matrix degradation. It is likely that the increased c-di-GMP levels present in the dt*amrZ* mutant strains impede matrix degradation, considering that biofilms by dt*amrZ* have been reported to harbor elevated levels of c-di-GMP, relative to those of wild-type biofilms, and that AmrZ represses the diguanylate cyclase-encoding gene *gcbA* (PA4843) (67). As for additional factors, our study identified two genes not previously linked with the response by P. aeruginosa, namely, *napB* and PA1891. This is supported by the finding that *napB* and PA1891 are expressed in an AmrZ-dependent manner (Table 1; Fig. 7A), and that biofilms formed by transposon insertional mutants of *napB* and PA1891 were deficient in dispersion and/or demonstrated reduced dispersion in response to nitric oxide (Fig. 7), relative to wild-type biofilms. Moreover, our findings indicated PA1891 not only to be dependent on AmrZ but also to be affected by BdlA (Fig. 8; Table 1). Our findings are in agreement with those previous reports of AmrZ affecting the transcript abundance of PA1891 and binding approximately 1,800 bp upstream of the first gene in the operon containing PA1891 (71). PA1891, encoding a hypothetical protein, is part of a 7-gene operon (83). None of the genes comprising this operon, including PA1891, have previously been characterized or harbor any conserved domains. However, PA1891 has been predicted to be localized in the membrane (83). More is known about NapB. The gene is part of the *nap* operon, comprising a total of 6 genes. *napB* is the 5th gene in the operon, with the last gene being *napC*.

Interestingly, AmrZ ChIP-seq data revealed that AmrZ binds within 300 bp downstream of *napC*, with a fold enrichment of 17.44 for this region (67). *napB* encodes a cytochrome c type protein NapB precursor. *napB* is part of the *napEFDABC* operon, which encodes genes that code for the periplasmic nitrate reductase complex Nap (83). In P. aeruginosa, Nap is one of three known nitrate reductases that are utilized for growth in nutrient-limited and oxygen-limited environments (84). In particular, the lung microenvironment has been reported to activate the expression of *nap* along with the denitrification operons *nor*, *nir*, *nar*, *nos*, leading to the expression of terminal oxidases with a high affinity to oxygen and a strong induction of a putative thiosulfate reductase-encoding operon (85, 86). While denitrification has not been directly linked to dispersion, nitrate has been shown to act as the best nitrogen source for the production of the biosurfactant rhamnolipids, with the exogenous addition of purified rhamnolipids to wild-type biofilms coinciding with the disassembly of the biofilm structure (87, 88). While our findings suggest a potential link between denitrification and dispersion, it is important to note, however, that the biofilm growth medium used in the current study, VBMM, does not contain nitrate, but instead contains ammonium chloride. Therefore, further studies on the role of *napB* and the *nap* operon are needed in order to define a more clear role for this gene/operon in dispersion. Regardless of the function of *napB* and PA1891, our findings strongly suggest that PA1891 and *napB* are the main contributors to the dispersion response (Fig. 7 and 8) and are the likely reason for the impaired dispersion response by dtamrZ strains (Fig. 9).

Collectively, our data indicate that AmrZ not only isthe central regulator of biofilm formation by P. aeruginosa (67, 89, 90) but also plays a pivotal role in the dispersion response by P. aeruginosa biofilms. In addition to being required for dispersion to occur, our findings further suggest that AmrZ functions downstream of BdlA. Moreover, our study resulted in the identification of two factors, NapB and PA1891, to be important for environmentally induced and BdlA mediated dispersion. The phenotype of biofilms formed by the *napB*::IS mutant suggested AmrZ-dependency, whereas the dispersion phenotype of PA1891::IS mutant strains suggested that, like AmrZ, PA1891 acts downstream of BdlA. Considering that we were unable to induce dispersion by biofilms formed by dt*amrZ* upon the overproduction of the endonuclease 1 EndA (41) or hydrolases PelA and PslG (40), and with both *endA* and *pelA* being directly regulated by AmrZ (67), our findings further indicate that AmrZ contributes to the regulation of additional factors, in addition to matrix degradation, that are essential for dispersion. An additional layer of complexity is introduced by the ability of AmrZ to function as a repressor and as an activator, reciprocally regulating genes that are essential for dispersion, such as *pelA* and *gcbA*.

The main interest of the paper is generated by (i) the finding that *amrZ* is required for dispersion and is downstream of BdlA and (ii) the identification of *napB* and PA1891 as important for NO mediated and BdlA mediated dispersion, respectively. The phenotype of the PA1891 mutant is particularly interesting, suggesting that, like AmrZ, it acts downstream of BdlA.

## MATERIALS AND METHODS

### Bacterial strains, plasmids, media and growth conditions.

The bacterial strains and plasmids used in the present study are listed in Table 2. The PAO1 transposon mutants were obtained from the sequence-verified two-allele library (91). Pseudomonas aeruginosa PAO1 was utilized as the parental strain for all of the experiments. Planktonic cultures were grown in Lennox broth (LB) or Vogel and Bonner citrate minimal medium (VBMM) at 37°C and 220 rpm. Biofilms were grown as indicated below. Antibiotics for plasmid maintenance were used at the following concentrations: 250 μg/mL carbenicillin and 50 to 75 μg/mL gentamicin for P. aeruginosa and 100 μg/mL ampicillin and 20 μg/mL gentamicin for E. coli. Arabinose was added to the growth medium at a concentration of 0.1 or 1% to induce gene expression in biofilms where indicated.

**TABLE 2:** Strains and plasmids used in this study

Strain/plasmid	Relevant genotype or description	Source
Strains		
Escherichia coli		
DH5α	*F^−^φ80lacZΔM15 Δ(lacZYA-argF)U169 recA1 endA1 hsdR17(rk^−^, mk^+^) phoA supE44 thi-1 gyrA96 relA1 tonA*	Life Technologies
BL21	F*^−^ ompT hsdS*_B_ (r_B_-m_B_-) *gal dcm rne131 (DE3)*	Life Technologies
P. aeruginosa		
PAO1	Wild-type strain PAO1	B.H. Holloway
*napB*::IS	PAO1; *napB*::*phoA*; Tet^R^	83
PA1891::IS	PAO1; PA1891::*lacZ*; Tet^R^	83
dt*amrZ*	WFPA205; PAO1 dtamrZ::tet; *amrZ* replaced with omega tetracycline cassette; Tet^R^	67
*amrZ*::Tet *attB*::P_BAD_-*amrZ*	WFPA203; PAO1 *ΔamrZ*::*Tet attB*::pBAD-*amrZ*; wild-type *amrZ* replaced with omega tetracycline cassette and arabinose inducible *amrZ* inserted at attB site; Tet^R^	67
Plasmids		
pJN105	Arabinose-inducible gene expression vector; pBRR-1 MCS; *araC*-P_BAD_; Gm^R^	97
pJN-*bdlA*-G31A	Arabinose-inducible expression of C-terminal 6xHis-tagged *bdlA* with G31A mutation cloned into pJN105, Gm^R^	27
pJN-PA1891	PA1891 cloned in to pJN105 at *SacI/NheI*, Gm^R^	This study
pMJT-1	Arabinose-inducible gene expression vector; pBRR-1 MCS; *araC*-P_BAD_; Gm^R^	98
pMJT-*napB*	*napB* cloned in to pMJT1 at *SacI/NheI* restriction sites, Carb^R^	This study
pHERD20T	Arabinose-inducible gene expression shuttle vector; Carb^R^	99
pHERD-*amrZ*	*amrZ* cloned into pHERD20T at *Xba*I/*Hin*dIII; *amrZ* harbors C-terminal 6x His tag; Carb^R^	67
CTX-P_bdlA_-*bdlA*-V5/His	V5-6xHis-tagged *bdlA* with native promoter cloned into mini-CTX, Tet^R^	12

### Strain construction.

*napB* and PA1891 were amplified using the primers listed in Table 3 and cloned into pJN105 or pMJT-1 at the sites indicated in Table 2. All of the plasmids were introduced by conjugation or by electroporation. Plasmid inserts were verified via DNA sequencing. Mutant strains were verified using the primers listed in Table 3.

**TABLE 3:** Oligonucleotides used in this study

Oligonucleotide	Sequence and purpose
Cloning	
pJN105_MCS_F	TAGCGGATCCTACCTGACGC
pJN105_MCS_R	CCATTCGCCATTCAGGCTG
pMJT1_MCS_F	GACCGCGAATGGTGAG
pMJT1_MCS_R	GAGCTGATACCGCTCG
*napB*_NheI_Cloning_for	GCGCGCGCGCTAGCATGAAACCTCTGCTGACT
*napB*_sacI_Cloning_rev	GCGCGCGCGAGCTCTTCATGCGGCCTCCCTCA
PA1891_NheI_Cloning_for	GCGCGCGCGCTAGCATGAGCGGACTCGCG
PA1891_SacI_Cloning_rev	GCGCGCGCGAGCTCGCGGGGGCGCCGGCTA
Verification of deletion or transposon insertion	
PA1891_F	CAGCAGCGACCAGATCCT
PA1891_R	GCCCAGAGGGCGAAGTAG
*napB*_F	GCTATCGCATCGACAAGG
*napB*_R	ATTGGCGGCTTCTTTCTC
*amrZ*_F	ACTGAAACAGGCAACTCCTACC
*amrZ*_R	GCTCGTGCAGGCTGAGTT
qRT-PCR	
*cysD*_ qRT_F	CTGGACATCTGGCAATACAT
*cysD*_ qRT_R	TCTCTTCGTCAGAGAGATGC
*pslA*_qRT_F	CGCGACCAAACTGGTACAC
*pslA*_ qRT_R	CAGGCGGTTGCTGAAGATATC
*pelA*_ qRT_F	GGTGCTGGAGGACTTCATC
*pelA*_ qRT_R	GGATGGCTGAAGGTATGGC
*pslG*_ qRT_F	CACGTAAGGGACTCTATCTGG
*pslG*_ qRT_R	AGGAAGTCTTTCCAGACCAC
*eddA*_ qRT_F	CCGACCAGTCGATCTTCTA
*eddA*_ qRT_R	TCCAGACGAAACGGATATT
*endA*_ qRT_F	GCTTTCCCGTTTGTTTGT
*endA*_ qRT_R	TAGAGCTTCCAGCCGATT
*cdrA*_ qRT_F	CGAACATCAGCGACGAAC
*cdrA*_ qRT_R	GATCGACAGGCCATC
*gcbA*_ qRT_F	CATGGAAGAACTGGCCGAC
*gcbA*_ qRT_R	GTCCTTCAGTGCCAGGTAG
*amrZ*_ qRT_F	AACACCGAGATTGTCTTGC
*amrZ*_ qRT_R	ACTGAAACAGGCAACTCCTAC
*napB*_ qRT_F	TGATCAGCATCACCCACT
*napB*_ qRT_R	CTCGAGGATCTGGTCGAT
PA1891_ qRT_F	CTTCGGCCTGTACCTGTT
PA1891_ qRT_R	CCAGAGGGCGAAGTAGAG
PA2655_ qRT_F	GTGCTGGTGTTCCTGTTG
PA2655_ qRT_R	GCAACGCGTTTTCCA
PA2750_ qRT_F	GTGGCGATACATGACGAC
PA2750_ qRT_R	CGAGCAGCATGTCTTCC
PA2819_ qRT_F	AACCTGGATCATGTTTGGA
PA2819_ qRT_R	AAGTTGTAACGCGGGAAT
PA2933_ qRT_F	CTGTTCGTCCTGCTGATG
PA2933_ qRT_R	CAGGCGGAGATGTTCAG
*vreA*_ qRT_F	GCTGCAACTCTGGATCG
*vreA*_ qRT_R	CAGCAACAGGATGGTCAG
*vreR*_ qRT_F	GTGTTCAACGACGTACCG
*vreR*_ qRT_R	CAGTTGATCGAGGCTGAA

### Biofilm growth.

To extract RNA or evaluate dispersion, biofilms were grown for 5 days under continuous flow conditions in biofilm tube reactors (1 m long, size 14 silicone tubing, Masterflex, Cole Parmer, Inc.) with an inner surface area of (25 cm^2^ at a flow rate of 0.2 mL/min), using 5-fold diluted VBMM medium (4, 17). For plasmid maintenance, 8 μg/mL carbenicillin and 2 μg/mL gentamicin were added. Where indicated, the growth medium was supplemented with 0.1% arabinose to induce the expression of genes of interest. For the visualization of the biofilm architecture, the biofilms were grown in flow cells (glass surface, BioSurface Technologies) at a flow rate of 0.2 mL/min. Following 5 days of growth, the biofilms were viewed via confocal laser scanning microscopy (CLSM), using a Leica TCS SP5 confocal microscope. Prior to confocal microscopy, biofilms were stained using the *Bac*Light LIVE/DEAD viability stain (Life Technologies) at a 1/1,000 dilution in the growth medium. The CLSM images were processed using LAS AF software v2.4.1. The quantitative analysis of the images was performed using the COMSTAT software package (92).

### Biofilm dispersion.

Dispersion assays were performed using biofilms grown in tube reactors for 3 or 5 days. The dispersion of 5-day-old biofilms was induced by the sudden addition of l-glutamate (18 mM) or sodium nitroprusside (500 μM) to the growth medium, as previously described (11, 93). Sodium nitroprusside was used as a source of NO. In addition, biofilms were grown for 5 days in the presence of 1% arabinose to induce hyperdispersive conditions via *bdlA_*G31A gene expression. Regardless of the dispersion cue used, dispersed cells were collected from the tube reactor effluents into 96-well microtiter plates at 1 min intervals for a total of 35 or 90 min. The absorbance of the biofilm effluents was assessed by spectrophotometry at 600 nm. The effluent profile was subsequently assessed for sharp increase in the absorbance values, as dispersion events are apparent by sharp increases in the absorbance values (600 nm) in the effluents of biofilm tube reactors, with the absorbance being at least two times greater than the that of the baseline of untreated biofilms or of those of the respective vector controls or nondispersive controls (27, 40, 41). Dispersion events in response to dispersion cues (glutamate, nitric oxide) have been reported to occur within 15 to 20 min upon the induction of dispersion, compared to untreated or control biofilms, and within 30 to 90 min upon the induction of gene expression (27, 40, 41). Therefore, we only considered sharp increases in the absorbance values to be indicative of dispersion within the indicated time frames and when the overall absorbance value exceeded that of the controls.

### RNA extraction and quantitative reverse transcriptase PCR (qRT-PCR).

To obtain RNA from the biofilms, wild type and mutant strains were grown in biofilm tube reactors in 5-fold diluted VBMM medium. Where indicated, the growth medium was supplemented with 0.1% arabinose to induce the expression of genes under the control of the pBAD promoter. Following 5 days of growth, the biofilm cells were collected directly into equal volumes of RNA Protect (Qiagen). The isolation of mRNA and cDNA synthesis was carried out as previously described (94–96). qRT-PCR was performed, using the Bio-Rad CFX Connect Real-Time PCR Detection System (Bio-Rad) and SsoAdvanced SYBR Green Supermix (Bio-Rad) with the oligonucleotides listed in Table 3. *cysD* was used as a control. Relative transcript quantitation was accomplished by first normalizing the transcript abundance (based on the threshold cycle value [Ct]) to *cysD* and then determining transcript abundance ratios. Melting curve analyses were employed to verify specific single product amplification.

### Immunoblot analysis.

The abundance and processing of tagged BdlA constructs were assessed via SDS-PAGE and immunoblotting, using anti-V5 antibodies. Total protein cell extracts (30 μg) were separated by SDS-PAGE and assessed via immunoblot analysis for the presence of V5/His tagged BdlA protein, using anti-V5 antibodies (Invitrogen Corp.). The antibodies were used at 0.1 μg/mL.

### Statistical analysis.

For the pairwise comparisons, a two-tailed Student's *t* test. assuming equal variances, or a single-factor analysis of variance (ANOVA) was used. In addition, the statistical differences between strains and/or conditions were determined via a one-way ANOVA, and this was followed by Dunnett’s *post hoc* test, using Prism5 software (Graph Pad, La Jolla, CA, USA). Unless otherwise noted, all experiments were performed at least in triplicate, using biological replicates.
